# 

**Published:** 1882-12

**Authors:** 


### INDEX TO VOLUME XLV.

PAGE.

A.

Abscess of Brain, J.T. Eskridge 291
Abstracts....................108 282
Aboriginal American Literature.................... 671
Actual Cautery in Uterine Cancer, Stroinski................ 476
Age of American Colleges..... 240
Agnew, Principles and Practice of Surgery, Review.......... 407
Agoraphobia, Potter........... 472
A Gubernatorial Doctor....... 671
Allen, System of Human Anatomy, Review................ 612
Allen, J, Adams............198 447
Amaurosis Chinica........... 420
American Dermatological Association.................481, 585
American Medical College Association ................. 63
American Public Health Association.................... 260
American Medical Association, Transactions, Review ...... 401
Amnesic Aphasia, E. Wyllys Andrews .................. 250
Anæsthetics, Manageable Zone of......................... 446
Anæsthesia of Plants........... 427
Andrews, E., Suction Drainage Tube ....................... 469
Andrews, E. Wyllys, Amnesic Aphasia, Case............... 250
Aneurism of Aorta............ 275
Anthropological Society, German.......................... 671
Antiseptic Cologne............ 533
Antiseptic Surgery, Park ..... 337
Aphonia in Singers and Orators 61
Archæology................... 668

PAGE.

Arsenic, Action of in Skin Diseases ..................... 201
Artificial Respiration........ 69
Atkinson, Vomiting in Pregnancy...................... 661

B.

Baby Incubators.............. 670
Bacillus Tuberculosis, Microscopical Investigation of, Schmidt..................... 561
Bacilli of Tubercle........... 429
Bacillus Tuberculosis.......... 408
Bailey S., Death from Thirty Grains of Dover's Powder... 26
Balfour, Professor F. M., Promotion of.................. 64
Balfour, F. M................ 671
Beale, New Materialism, Review........................ 615
Belfield, W. T., Vienna Letters. 37 170
Bern, Law Enforcing Vaccination ...................... 240
Bettman, Jefferson, Laryngeal Hæmorrhage............... 142
Bile and Digestion, Stokes..... 313
Biological Society............. 518
Books and Pamphlets......276 617
Bliss, Dr..................... 446
Blood, A Study of, Lester Curtis ........................ 65
Bowel Affections of Young Children, N. S. Davis.......... 15
Brower, D. R., Paper on Toy Pistol Tetanus.............. 449
Burial Permits, Ordinance in Relation to................ 164
Byford, H. T................ 670
Byford, Wm. H., Diseases of Women, Review............ 376
Byford, W. H................ 198

PAGE.

C.

Carr, J. D. M., Death of....... 670
Canine Appreciation.......... 281
Cancer of the Ileum........... 280
Carbolic Acid Poisoning...... 553
Cathell, W. D, The Physician Himself, and What He Should Add to the Strictly Scientific. 194
Cautery in Uterine Cancer, Stroinski.................. 476
Chicago Medical College...... 375
Chicago Medical Society....48 154
Cholera in Japan............ 670
Chapman, N. H., Dorso Lumbar Abscess................ 438
Chronic General Diseases, Prof. N. S. Davis................ 225
Chicago Pathological Society 159 516
Cholera in West Africa.... ... 110
Chinoline in Diphtheria. . ... 554
Chronic general Diseases, N. S. Davis .................... 225
Chronic Urethritis, Rare Incidents in.................... 546
Chronic Cystitis and Operation for Polypus of the Bladder, Stroinski.................. 478
Cirrhosis of Pancreas, Two Cases, C. W. Earle.......... 254
Clark, H. H., New Suture Button........................ 27
Clinics .. 112, 224 336 448 560 672
Clinical Reports........... .. 32
Clinical Study of Small Granular Cells of the Blood, Davidson ................... ... 324
College of Physicians and Surgeons .... ............... .. 375
Congress of Hygiene.......... 553
Congressional Appropriations for Medical Purposes, Editorial........ ............. 62
Contagion of Yellow Fever.... 272
Contagious Diseases of Domestic Animals, E. M. Hunt. ... 78
Contusion of Popliteal Artery, Death ..................... 203
Congress of German Surgeons.. 203
Congenital Double Luxation of the Femur................. 550
Cool Air in Measles and Scarlet Fever..................... 285
Cook County Hospital........ 274
Correspondence, Domestic, 45, 40 272 271 179 46 522 611
Correspondence, Domestic, Immigrant Inspection Service, Michigan State Board of Health ......... .......... 178

PAGE.

Correspondence, Foreign....37, 263, 267, 170, 173
Cremation in Milan........... 538
Curtis, Lester, Fallacies of the Bioplasson Theory.......... 430
Curtis, Lester, Study of Blood. 65
Curtis, Lester, S phæro Bacteria in Blood of Tetanus.......... 462

D.

Dalton, J. C., Treatise on Physiology, Review ...... ...... 404
Danforth, I. N., Valedictory Address to Graduating Class. 21
Dana, C. L., New York Letters, 522, 529
Davidson, Study of the Small Granular Cells of the Blood.. 324
Davis, N. S., Bowel Affections of Young Children, Prevention of..................... 15
Davis, N. S., Chronic General Diseases................... 225
Davis, E. P., Epileptiform Neuralgia, Report............... 32
Death From Thirty Grains of Dover's Powder, Dr. S. Bailey 26
Deflection of Septum Narium, E. F. Ingals................. 627
Demokedes, of Kroton ........ 53
Dental Infirmary..........669, 671
De Wolf, Glycosuria........... 642
Diet Cure, The, Nichols, Review......................... 189
Diseases of Women, Byford, Review.................. ... 376
Diseases of Children, Meigs, Review. .................... 404
Divorce in Mental Alienation.. 201
Doctors vs. Politics........... 52
Doctor, What Shall I Eat? Gatchell, Review................ 182
Domestic Correspondence, 40, 45, 46, 271, 522, 611
Donaldson, Practical Gynæcology, Review................ 616
Dorso Lumbar Abscess, Treatment of, Chapman ......... 438
Drainage of Echinococcus Cyst 545
Drainage Tube for Suppurating Pleural Cavities, Andrews... 469
Dredging Expedition in Bay of Biscay. ................... 47
Drugs That Enslave, Kane, Review...................... 187
Dudley, E. C.......... ...... 198
Dwight, Thos., Frozen Sections of a Child................. 405
Dystocia from Transverse Occlusion of Uterus......... . 180

PAGE.

E.

Earle, C. W., Cirrhosis of Pancreas, Cases.............. 254
Editorial..62, 196, 274, 408, 534, 620
Egypt as a Health Resort...... 669
Electricity, The Cause of Death. 447
Elgin Letter, Rosencrans...... 532
Embalming.................. 551
English Criticisms of Americans...................... 620
Epidemic Cholera......... ... 447
Epidemics, Actual or Threatened..................... 334
Epidemic Cerebro-Spinal Meningitis..................... 108
Epileptiform Neuralgia, Clinical Report, E. P. Davis....... 32
Epilepsy with Mania, Caused by Masturbation............ 147
Erysipelas, Treatment of, G. F. Lydston.................... 1
Eskridge, Cases of Cerebral Abscess.................... 291
Ethics, The New York Code... 196
Euphorbia Pilulifera.......... 554
Explosions of Sewer Gas...... 670
Experimental Method in Medical Science, Notice, Dalton.. 617
Explanation.... ............. 291
Extirpation of the Larynx..... 278

F.

Fallacies of the Bioplasm Theory, Curtis. ............... 430
Fenn, C. T., Insanity. ......... 154
Fitch, T. D., Notice........... 669
Foster, Text-Book of Physiology Review.... ............ 185
Flint, Austin, Jr., Text-Book of Physiology, Review.......... 402
Formulary, A Medical, Johnson, Review............. ... 193
Foreign Correspondence....... 170, 173 263 267
Food for the Invalid, Fothergill, Review ...... ........ 192
Footprints of Man............ 671
Frozen Sections of a Child, Dwight, Review......... .. 405
Funerals, Expense of.......... 538

G.

Gastric Dyspepsia, W. Henry 29
Gatchell, Doctor what shall I eat? Review.. ............. 182
Grafting of Dead Upon Living Bone...................... 61
Griffin, B. W., Sewer Gas and Its Effects Upon Health...... 241

PAGE.

Gill, H. Z., Penitentiary Reports..................... 356
Glycosuria, DeWolf........... 642
Goding, F. W., Letter From Tama City..................... 46
Gunshot Wounds of the Abdomen and Modern Peritoneal Surgery, J. Marion Sims, M.D., 95
Gynæcology, Practical, Donaldson, Review................ 616

H.

Hagenbach, A. W., Hereditary Insanity .................. 113
Henry, Wm., Pernicious Anæmia..................... ...... 152
Henry, Wm., Gastric Dyspepsia, 29
Hereditary Insanity, Hagenbach....................... 113
Heredity .. ................. 59
Herpes, Niger, of Labia Majora, 169
Hillingham, Diseases of Rectum, Review............ ... 614
Histology, Manual of, Prudden, Review.......... ....... 616
Holmes, E. L., Severe Injury to Orbit..................... 555
Holmes, System of Surgery, Notice....................... 407
Homœopathy in Cook County Hospital. ................. 669
Hopkins, Tenosynovitis........ 332
Hospital Reports.............. 369
Hospital at Hot Springs, Ark.. 671
Hotz, J. C., Yellow Oxide of Mercury.... .............. 180
Hunt, Ezra M., Contagious Diseases of Domestic Animals.. 78
Huse, E. C., Hypodermic Medication...................., 271
Huse, E. C., New Stethoscope.. 372
Howard, Insanity and Imbecility, Review................ 406
How We Fed the Baby to Make Her Healthy and Happy, With Health Hints, Review.. 184
Hyde, James Nevins, Change of Location.................. 198
Hydrops Sinus Frontalis...... 550
Hypodermic Syringes......... 668
Hypodermic Medication....... 271
Hysteria in the Male.......... 57

I.

Icterus Neonatorum.......... 109
Illinois State Board of Health.. 162
Impotentia Viris.............. 56

PAGE.

Immigration and Disease Report, National Board of Health ...................... 256
Incidental Effects of Drugs, Lewin, Review ............. 190
Incubation of Communicable Diseases, B. W. Richardson 103, 205
Insanity in Australia .......... 669
Insanity, C. T. Fenn .......... 154
Ingals, E. F., Deflection of Septum Narium ................ 627
Inhalation of Medicated Vapor. 282
Injury to Orbit, Holmes and Park ...................... 555
Insanity in the U. S. Statistics.. 445
Insanity and Imbecility, Howard, Review ................ 406
Insanity and Divorce........111, 284
Insanity as Ground for Divorce 110
Interstitial Chronic Hepatitis.. 428
Iodoform Insanity........ .... 64
Iron as An Analeptic...... ... 111
Isham, R. N., Rupture Left Kidney..... ..... ........256 364
Items...................64, 444 668

J.

Japanese Medical Works in 1881 ......... .......... 671
Jenks, Edw. W................ 368
Jewish Military Physicians.... 553
Journal of the American Medical Association............. 401

K.

Kane, H. H., Drugs that Enslave, Review ................... 137
Keating, John M., Micrococcus in Blood of Malignant Measles.......... ....... ... 209

L.

Letters From Paris, R. Tilley, 263 267
Laryngeal Hæmorrhage, Jefferson Bettman. ....... ...... 142
Lectures on Electricity, Rockwell, Review..... ... ....... 182
Letter of French Ambassador at Berlin................... 426
Letters From Tama City, Gooding .................. . 46 531
Lingual Nerve, Effects of Excitation ........ .......... 549
Local Societies............ ... 513
Lydston, G. F., Treatment of Erysipelas.......... ....... 1

PAGE.

M.

Mallock, Social Equality, Review of.................... 613
Marriage Laws....... ... ... 669
Materialism, Beale, Review.... 615
Malaria on Man, Effects of..... 108
Manual of Midwifery, Meadows, Review............ ....... 184
Medical Profession in United States ...................... 670
Medical Directory ........ ... 443
Medical Heresies, Smythe, Review ...................... 186
Medical Troubles in Maine.... 531
Medical Society of Berlin. .... 554
Meigs, J. F., Diseases of Children, Review .............. 404
Merriman, Prof., H. P......... 198
Michigan State Board of Health 165
Micrococcus, The, in Malignant Measles, Keating............ 209
Micro-Organisms in Blood of Tetanus, Curtis............. 462
Mild Surgery............ ... 554
Murrell, Nitro-Glycerine, Review...................... 400
Muscular Atrophy............ 424
Myelitis, Case of.......... .... 412

N.

New York Letters ......40, 522 529
Notes From Private Practice. 148
Nitro-Glycerine as a Remedy, Murrell, Review... ....... 400
Nitrous Oxide................ 429
New Practitioners......... ... 444
Nerve Stretching......... .... 369
National Conference of Charities and Corrections.. ....... 273
National Hygienic Congress of Argentine Republic.. ... ... 554
Nature of Insanity........... 64

O.

Obituary.................... 559
O'Connell, P., Syphilitic Cases in Private Practice.......... 148
Ointment, Yellow Oxide Mercury, F. C. Hotz............ 180
One Kidney ................. 552
Opium Dens................ 447
Opium Smoking in America and China, Kane, Review.... 194
Opthalmological Clinics at Paris...................... 199
Organic Materia Medica, Conspectus of, Sayre. Review... 191

PAGE.
Organic Materia Medica, Manual of, Maisch, Review...... 183
Original Translations, O. Stroinski..................288, 418
Original Translations, Boss.... 412
Original Communications..241, 561
Osteotomy in Club Foot....... 546
Oxidized Water vs. Living Organisms.................... 111

P.

Park, Roswell, Antiseptic Surgery.......................... 337
Park, Roswell, Prag Letter.... 173
Park, Roswell, Severe Injury to Orbit, Report of............ 555
Pathological Society........... 516
Pelletierinum Tannicum...... 262
Pernicious Anæmia, Wm. Henry.......................... 152
Perforation of Œsophagus..... 544
Philosophical Society.......... 554
Physiology, a Treatise on, Dalton, Review................ 404
Physiology, a Text-Book of, Flint, Review............... 402
Physiology, a Text-Book of, Foster, Review.............. 185
Pilocarpine in Hydrophobia... 57
Pilocarpine in Poliuria....... 552
Pneumonia, Geography and Climate of, E. Sanders...... 289
Poisoned Cake.................. 521
Post Graduate School of Medicine.......................... 111
Post-Mortem of Guiteau....... 73
Potter, E. L., Agoraphobia..... 472
Potter, Speech and Its Defects, Review...................... 613
Practice of Medicine in Michigan, Regulation of........... 653
Prag, Letter, Roswell Park ... 173
Premature Labor from Quinine, Theodore Turnbull.... 179
Prolapsus of Colon............ 548
Prudden, Manual of Practical Histology, Review.......... 616
Pulmonary Orifice, Contraction of.......................... 428
Pyometra.................... 421

Q.

Quarantine in Egypt........... 63
Quackery, Definition of........ 277
Quinine in Cholera Infantum.. 287

R.

Reception to Francis Seymour Haden........................ 6 9

PAGE.
Rectum, Diseases of, Hillingham, Review.............. 614
Report from Penitentiary at Chester, H. Z. Gill.......... 356
Report of Post-Mortem Examination of the Body of Charles J. Guiteau................. 73
Resection of the Stomach..418, 554
Reviews and Book Notices 181, 376 612
Richardson, Incubation of Communicable Diseases...103, 205
Roberts, Relation of Syphilis to Scrofula..................... 329
Rosencrans, Contagion of Yellow Fever.................... 272
Rosencrans, Letters from Elgin..........................45, 532
Rupture of Stomach.......... 670
Rush Medical College........ 368
Rupture of Left Kidney, R. N. Isham, M.D..............256, 364
Rupture of Uterus............ 539
Ruskin, Paragraph From...... 204

S.

Sanders, E., Geographical Relations of Pneumonia........ 289
Sayre, Organic Materia Medica, Review.................... 191
Scarlatina, Complicated Case of, L. C. Waters............... 255
Sarcoma of Testicle........... 551
Scarlet Fever................. 671
Scientific Diagnosis of Disease. 111
Schmidt, Notice of, Article.... 625
Schmidt, Microscopical Investigation of the Bacillus Tuberculosis.................. 561
Seeley, W. W., Yellow Oxide of Mercury................... 351
See, Prof. Germain, on Convallaria Majalis............... 240
Selections, 65, 205, 291, 430, 555 627
Sensation and Pain, Taylor, Review..................... 181
Sewer-Gas, and Its Effects Upon Health, B. W. Griffin........ 241
Sewer....................... 611
Sex and Race................. 667
Siberian Plague.............. 447
Sign of Death................ 411
Sims, J. M., Gunshot Wound of Abdomen, in Relation to Modern Peritoneal Surgery.. 95
Society Reports, 48, 154, 254, 373 585
Social Equality, Review....... 613
Society of Physicians and Surgeons of Minneapolis....... 533
Speech and Its Defects, Review 613

PAGE.
Sphæro-Bacterium, the Latest, A Good Thing to Have in the Family.... ............... 534
State Board of Health of West Virginia.................... 635
Statistics of Insanity in the U. S., Dana..... ............... 445
Stebalts, Tetanus.............. 367
Stethoscope, New, E. C. Huse.. 372
Stokes, J. W., Bile and Digestion ...................... 313
St. Petersburg................ 56
Stretching of Median Nerve... 552
Stroinski, O., Chronic Cystitis and Laceration of the Bladder 478
Stroinski, O., Acual Cautery in Uterine Cancer............. 476
Stroinski, O., Original Translations from Foreign Exchanges....... ............... 278
Sudden Death in Phthisis..... 56
Suggestive.................... 626
Sunstroke, Discussion on...... 159
Supervised Prostitution....... 619
Surgery, System of, Gross, Notice......................... 617
Surgery, System of, Holmes, Notice .................... 407
Surgery, Principles and Practice, Agnew, Review........ 407
Suture Button, Dr. H. H. Clark. 27
System of Human Anatomy, Allen Review............... 612
Syphilis of Brain............. 423
Syphilis, Its Relation to Scrofula, Roberts.... ........... 329
Syphilitic Cases, P. O'Connell. 148

T.

Tansy, Poisoning by........... 46
Tertiary Syphilis.............. 541
Tenosynovitis, Hopkins....... 332
Tetanus, Nerve-Stretching in .. 369
Tetanus, Stebalts.............. 367
The Physician Himself, Cathell, Review..................... 194
Tilley, R., Letters from Paris, 263 267
Translations...........199, 539 53
Transactions American Medical Association, Review......... 401
Transmission of Nerve Force in Men...................... 111

PAGE.
Transfusion of Blood into the Peritoneal Cavity........... 551
Troubles in Cook County Hospital....... .............. 670
Tri-State Medical Society.. 169 261
Thompson, on Diseases of Urinary Organs, Review........ 615
Tuberculosis, Etiology of..... 55
Turnbull, Theodore, Premature Labor from Quinine..... ... 179

U.

Urinary Organs, Lectures on Diseases of, Thompson, Review...................... 615
University of Sidney.......... 626
Umbilical Hernia, Rupture of. 425
U. S. Dispensatory............ 668

V.

Vaccination in Switzerland.... 240
Valedictory Address to Graduating Class of Women's Medical College, I. N. Danforth.. 21
Vertigo de Meniere ... ...... 545
Vienna Letters, W. T. Belfield, 37, 170
Voisin, Dr. August, Origin of Suicidal Ideas............... 270
Vomiting in Pregnancy, Atkinson ........................ 661

W.

Walls of Houses.. ............ 670
Waters, L. C., Complicated Case of Scarlatina............... 255
Wilson, O. E., Obituary........ 559
Wedl, Prof. C............. ... 195
Woehler, Fredrich, Death of... 671
Woman's Medical College .... 375
Woman's Hospital, State of Illinois ....... ....... ..... 153
Worms from the Black Bass.... 447
Whooping Cough, Treatment of.......................... 60

Y.

Yellow Fever, Contagion of, H. Rosencrans.... ........ 272
Yellow Oxide of Mercury, Seeley ........ ............ 351
Yellow Fever................. 407

### BOOKS AND PAMPHLETS RECEIVED.

### BOOKS.

Ninth Annual Report State Board of Health of Michigan, Year Ending September 30, 1881. Lansing, W. George & Co. 1882.

Index Catalogue Library Surg. General's Office U. S. A. Washington, Government Print. 1882.

A Manual of Hypodermatic Medication. Bartholow. Phila., Lippincott & Co. 1882.

Asthma. Pathology and Treatment. Salter. Wood's Med. Lib. 1882. Keener.

Social Equality. A Short Study in a Missing Science. Mallock. New York, Putnam's Sons.

Science and Practice of Medicine. Palmer. Vol. II. G. P. Putnam's Sons W. T. Keener.

Diseases of the Rectum. Allingham. Blakiston, Son & Co. 1882.

Allen's Human Anatomy, Sections One and Two.

International Encyclopœdia of Surgery. Wm. Wood & Co. W. T. Keener.

St. Bartholomew's Hospital Reports, Vols. XVI and XVII. 1880 and 1881. Smith, Elder & Co. London.

Nitro-Glycerine as a Remedy for Angina Pectoris. Murrell. Davis. Detroit.

Therapeutics, Materia Medica and Toxicology. Wood. Fourth Edition.

System of Surgery. Gross. Vols. I and II. Phila., Lea's Son & Co. Jansen, McClurg & Co.

Pharmacopœia of the United States. Sixth Decennial Revision. 1882. Wm. Wood & Co. W. T. Keener, Chicago.

Rheumatism, Gout, and Some Allied Disorders. Longstreth. New York, Wm. Wood & Co., Lib. 1882. Chicago, W. T. Keener.

Anatomical Technology as Applied to the Cat. Wilder and Gage. Chicago, A. S. Barnes & Co.

The Uterus, Ovaries and Fallopian Tubes. Courty. Translated by McLaren. Phila., Blakiston, Son & Co. Chicago, W. T. Keener.

The Diseases of Women. Hewitt. Fourth Edition. Phila., Blakiston, Son & Co. Chicago., W. T. Keener.

Practice of Medicine. Bartholow. New York, D. Appleton & Co. Chicago, Jansen, McClurg & Co.

Hand-Book for Water-Drinkers. Austin. Boston, Lee & Shepherd. Chicago, Jansen, McClurg & Co.

Guide to Therapeutics and Materia Medica. Farquharson. Phila., Lea's Son & Co. Chicago, Jansen, McClurg & Co.

Vaccination and Small-Pox. Hardaway. Chicago, Jansen, McClurg & Co.

Speech and Its Defects. Potter. Phila., Blakiston & Son. W. T. Keener.

Practical Laboratory Course in Medical Chemistry. Draper. Wm. Wood & Co. W. T. Keener.

Slight Ailments, Their Nature and Treatment. Beale. Phila., Blakiston. W. T. Keener.

Medical Electricity. Bartholow. Second Edition.

King's Manual of Obstetrics. Jansen, McClurg & Co.

Diseases of the Ear in Children. Troeltsch. New York, Wm. Wood & Co. Chicago, Jansen, McClurg & Co.

Diseases of the Skin. Duhring. Third Edition. Phila., Lippincott & Co. Chicago, Jansen, McClurg & Co.

Principles and Practice of Surgery. Ashurst. Third Edition. Phila., Lea's Son & Co. Chicago, Jansen, McClurg & Co.

Cornil on Syphilis. Translated by Simes & White. Phila., Lea's Son & Co. Chicago, Jansen, McClurg & Co.

First Aid to the Injured. Shepherd. New York, G. P. Peterson & Co. Chicago, Jansen, McClurg & Co.

Medical Register and Directory, City and County of Philadelphia, P. Blakiston & Son. Chicago, Jansen, McClurg & Co.

Microscopical Morphology. Heitzmann. New York, J. Vail & Co. Chicago, W. T. Keener.

---

SUPERVISED PROSTITUTION.—The average number of examinations of individual women made in England during the year 1881 in the several places subject to the Acts was 23.65; in 1880 it was 23.50. One thousand, three hundred and fifty nine women were registered for the first time during the year, but a total decrease of 3,056 has taken place since the Acts came into operation. The number of brothels was reduced during the year by 44, being a decrease of 833 since the Acts came into operation. During the year, 489 prostitutes are known to have come into the several districts from unprotected places; 322 of these were found to be diseased on their first medical examination. In 1881, 1,827 were removed from the register, of whom 863 left the districts under the protection of the Acts, 51 married, 234 entered homes, 659 were restored to friends, and 20 died.

MR. FRANCIS SEYMOUR HADEN, the distinguished member of the Royal College of Surgeons in Great Britain, who has attained a reputation as an etcher equal to that of Rembrandt, was recently given a reception in one of the Chicago clubs. He stated that his first achievements in his art were the fruits of the employment of his leisure moments in his practice. Speeches of welcome were made on the occasion by Dr. Chas. Gilman Smith, Dr. James Nevins Hyde, Mr. Ed. G. Mason, and others.

CLINICS.

MONDAY.

Eye and Ear Infirmary—1.15 to 2.15 p. m., Otological, by Dr. Schaefer; 2.15 to 3.30 p. m., Ophthalmological, Dr. Holmes.

Mercy Hospital—2 p. m., Medical, Profs. Hollister and Quine.

Rush Medical College—3 p. m., Dermatological and Venereal, by Prof. Hyde.

Woman's Medical College—2 p. m., Dermatological and Venereal, by Prof. Maynard.

TUESDAY.

Cook Co. Hospital—2 to 4 p. m., Medical and Surgical Clinics.

Mercy Hospital—2 p. m., Surgical Clinic, by Prof. Andrews.

Woman's Medical College—10 a. m., Prof. Ingals.

WEDNESDAY.

Chicago Medical College—2 p. m., Eye and Ear, by Prof. Jones.

Rush Medical College—2 p. m., Medical by Prof. Bridge; 3 p. m., Ophthalmological and Otological, by Prof. Holmes: 3 to 4 p. m., Diseases of the Chest, by Prof. Ross.

Woman's Medical College—2 p. m., Eye and Ear, by Dr. W. T. Montgomery; 3 p. m., Diseases of Children, by Prof. Chas. W. Earle.

Eye and Ear Infirmary—2.30 p. m.. Dr. E. J. Gardiner.

THURSDAY.

Chicago Medical College—2 p. m.. Gynæcological, Prof. Dudley.

Rush Medical College—2 p. m., Diseases of Children, by Prof. Knox; 3 p. m., Diseases of the Nervous System, by Prof. Lyman.

Eye and Ear Infirmary—1.15 to 2.25 p. m., Otological, by Dr. Schaefer; 2.15 to 3.30. p. m., Ophthalmological, Dr. Holmes.

Woman's Medical College—3 p. m., Surgical, by Prof. Owens.

College of Physicians and Surgeons—2 p. m., Medical, by Prof. S. A. McWilliams; 3 p. m., Surgical, by Prof. R. L. Rea.

FRIDAY.

Cook County Hospital—2 to 4 p. m., Medical and Surgical Clinics.

Mercy Hospital—2 p. m., Medical, by Prof. Davis.

SATURDAY.

Rush Medical College—2 p. m., Surgical, by Prof. Gunn.

Mercy Hospital—2 p. m., Surgical Clinic, by Prof. Andrews.

Chicago Medical College—3 p. m., Neurological, Prof. Jewell.

Woman's Medical College—2 p. m., Gynæcological, by Prof. Stevenson.

College of Physicians and Surgeons—2 p. m., Diseases of the Chest, by Prof. S. A. McWilliams; 3 p. m., Gynæcological, by Prof. A. Reeves Jackson.

Daily Clinics, from 2 to 4 p. m., at the Central Free Dispensary, at the South Side Dispensary and at the West Side Dispensary.

